# Paraneoplastic Neurological Syndromes Associated With Parotid Cancer Expressing the Zic4 Antibody: A Case Report

**DOI:** 10.7759/cureus.90210

**Published:** 2025-08-16

**Authors:** Masahiko Oyanagi, Akito Kakiuchi, Keisuke Yamamoto, Makoto Kurose, Kenichi Takano

**Affiliations:** 1 Otolaryngology-Head and Neck Surgery, Sapporo Medical University School of Medicine, Sapporo, JPN

**Keywords:** head and neck cancer, paraneoplastic neurological syndromes, parotid cancer, pns, zic4

## Abstract

Paraneoplastic neurological syndromes (PNS) are rare, immune-mediated disorders associated with cancer, often presenting with various neurological symptoms, such as encephalomyelitis, limbic encephalitis, and rapidly progressive cerebellar syndrome. A 66-year-old woman presented to our department with rapidly increasing swelling in her right parotid gland. Fine needle aspiration cytology confirmed parotid cancer, and a right parotidectomy and dermectomy were planned. Preoperatively, she experienced symptoms of nausea and vertigo and was later diagnosed with PNS based on the presence of Zic4 antibodies in the cerebrospinal fluid. Following methylprednisolone pulse therapy and tumor resection, the patient's symptoms resolved completely. Histopathological examination of the surgically excised tissues confirmed a diagnosis of salivary duct carcinoma. Immunohistochemistry revealed that the Zic4 antibody stained the nucleoli of tumor cells. This report presents the first documented case of PNS linked to parotid cancer expressing the Zic4 antibody, along with a review of 21 previous cases of PNS associated with head and neck cancers.

## Introduction

Paraneoplastic neurological syndromes (PNS) are rare immune-mediated disorders associated with cancer [1,2]. Over time, numerous antibodies associated with PNS have been identified, serving as diagnostic markers for underlying tumors. PNS manifests through various neurological symptoms, including encephalomyelitis, limbic encephalitis, rapidly progressive cerebellar syndrome, opsoclonus-myoclonus syndrome, sensory neuropathy, gastrointestinal pseudo-obstruction, Lambert-Eaton myasthenic syndrome, among others [3]. Although PNS is associated with various cancer types, such as small-cell lung, testicular, ovarian, and breast cancers, reports on its association with head and neck cancers are sparse [4]. Herein, we present the first reported case of PNS linked to Zic4 antibody-expressing parotid cancer and review 21 previous cases of PNS associated with head and neck cancers.

## Case presentation

**Figure 1:**
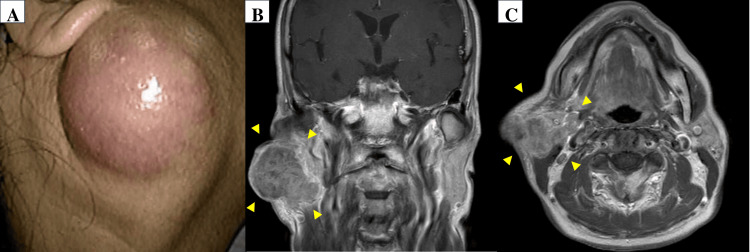
Right parotid tumor and enlarged level II lymph nodes (A) A mass lesion, approximately 45 mm in size, was identified within the right parotid gland. (B, C) Contrast-enhanced MRI of the head and neck showed a 45 mm × 38 mm tumor within the right parotid gland, demonstrating partial contrast enhancement and subcutaneous invasion; the parotid gland appeared fused with enlarged level II lymph nodes.

A 66-year-old woman presented to our department with rapidly increasing swelling in the right parotid gland, which had progressively enlarged over the past few years. Her medical history included ovarian cyst removal in her 20s and a 10-year history of smoking approximately 20 cigarettes daily. Upon examination, an approximately 45 mm mass was identified within the right parotid gland. The lesion exhibited skin erythema, a smooth surface, and mild pressure pain. No associated facial nerve palsy was observed (Figure 1A). Magnetic Resonance Imaging (MRI) with contrast enhancement of the head and neck revealed a 45 mm × 38 mm tumor with subcutaneous invasion, and fusion of the parotid gland with enlarged level II lymph nodes was noted (Figures 1B, 1C). No other lymph node metastases were observed. Fine-needle aspiration cytology of the parotid gland confirmed the presence of parotid carcinoma, though the detailed histology remained unknown. Fluorodeoxyglucose-positron emission tomography (FDG-PET) revealed no evidence of distant metastasis. Consequently, we diagnosed the patient with right parotid cancer and scheduled a right parotidectomy, including skin complication resection, right neck dissection, and reconstruction using an anterolateral thigh flap. Preoperatively, the patient was admitted to our department with subacute symptoms of nausea and vertigo, resulting in difficulty walking. Three days before admission, she had experienced vertigo triggered by body movements, particularly head movements, which made it difficult for her to walk. The vertigo symptoms had been gradually worsening. On examination, cognition was intact without spontaneous nystagmus. However, horizontal gaze nystagmus was observed, which was more pronounced on left gaze. Furthermore, a positive finger-to-nose test indicated significant coordination difficulties in the upper limbs, especially on the right side. Routine biochemical and hematological tests yielded normal results. Additionally, an MRI of the head revealed no signal changes in the brain parenchyma, particularly in the brainstem and cerebellum, where no abnormalities were observed.

**Figure 2:**
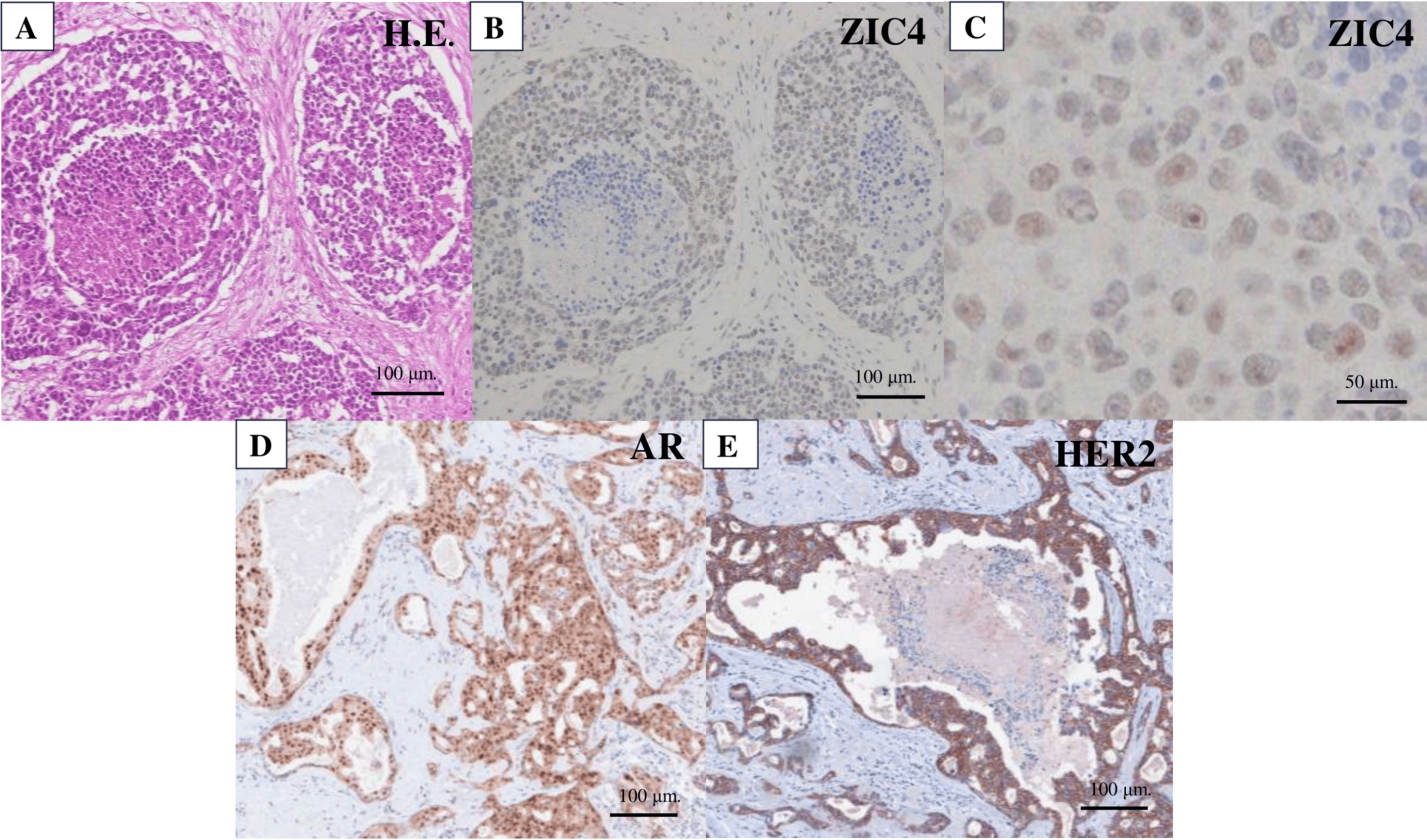
Pathology of parotid cancer (A) Hematoxylin and eosin (HE) staining revealed tumor cells with large round nuclei and tubular and cribriform structures with prominent comedo necrosis. Scale bar: 100 μm. (B) Immunostaining demonstrated that the tumor cells were positive for the Zic4 antibody. Scale bar: 100 μm. (C) Zic4 antibody immunostaining specifically stained the nucleoli of tumor cells. Scale bar: 50 μm. (D) Immunohistochemistry demonstrated that the tumor cells were positive for the androgen receptor (AR). Scale bar: 100 μm. (E) Immunohistochemistry demonstrated that the cells expressed human epidermal growth factor receptor 2 (HER2). Scale bar: 100 μm.

Computed Tomography (CT) of head imaging revealed no lesions that could account for the patient's symptoms. The cerebrospinal fluid (CSF) was clear, with a cell count of 6 cells/μL, glucose concentration of 91 mg/dL (compared to 118 mg/dL blood glucose), protein concentration of 56 mg/dL, and the presence of positive oligoclonal bands. CSF cytology showed no evidence of malignancy, and consecutive serum and CSF analyses did not indicate any active infection. Given the patient's neurological symptoms, test results, and the presence of parotid cancer, PNS was suspected. Neuronal autoantibodies were tested, revealing positivity for the Zic4 antibody in the CSF, while other antineuronal antibodies (AMPH, CV2, PNMA2, recoverin, SOX1, titin, Hu, Ri, Yo, GAD65, and Tr) were negative. Based on these findings, the patient was diagnosed with PNS. Treatment was initiated with intravenous methylprednisolone (1,000 mg/day) for five days. Although the patient’s symptoms improved, they did not resolve completely, and the planned radical tumor resection was performed. Postoperatively, the patient's symptoms resolved completely. Histopathological examination of the parotid cancer revealed tumor cells with large, round nuclei and tubular and cribriform structures, along with prominent comedo necrosis, as seen in hematoxylin and eosin (HE) staining (Figure 2A). Immunohistochemistry showed that the Zic4 antibody stained the nucleoli of tumor cells (Figures 2B, 2C). Additionally, the tumor cells expressed human epidermal growth factor receptor 2 (HER2) and were positive for the androgen receptor (AR) (Figures 2D, 2E).

These findings confirmed the diagnosis of salivary duct carcinoma (SDC). Subsequently, she underwent radiation therapy, receiving a total dose of 66 Gy in 33 fractions using intensity-modulated radiation therapy (IMRT). Six months post-surgery, metastases were detected in the left lung and multiple bones, including the sternum, ribs, and thoracolumbar spine. No local recurrences were observed. Metastatic disease was treated with chemotherapy and palliative irradiation. The chemotherapy regimen included docetaxel (8 mg/kg) and Herceptin (70 mg/m2). Radiotherapy was administered, delivering a total dose of 20 Gy in five fractions. Despite undergoing five courses of chemotherapy, the tumor remained uncontrolled, leading to the adoption of a palliative care approach. No cerebellar symptoms have been observed since the operation.

## Discussion

PNS is a rare immune-mediated disorder triggered by underlying tumors [1,2]. PNS occurs in > 0.1% of all patients with malignancy and is associated with various cancer types [4]. However, reports of PNS associated with head and neck cancers are sparse. To our knowledge, this is the first reported case of PNS due to parotid cancer.

PNS manifests with neurological symptoms, including encephalomyelitis, limbic encephalitis, rapidly progressive cerebellar syndrome, opsoclonus-myoclonus syndrome, sensory neuropathy, gastrointestinal pseudo-obstruction, Lambert-Eaton myasthenic syndrome, and others [3]. These symptoms sometimes precede tumor detection, offering an opportunity to identify undiagnosed tumors [5]. Approximately 50% of patients diagnosed with PNS exhibit neural antibodies that activate the immune system, leading to inflammation-mediated damage to neural tissues [1]. These antibodies bind to intracellular proteins expressed in the cytoplasm, nucleus, and nucleolus. While they do not directly cause neuronal damage, cytotoxic T cells are believed to be the primary effectors of neuronal injury [6]. In our patient, the Zic4 antibody was found to bind to the intracellular nucleolus of the parotid cancer cell, supporting this theory.

**Figure 3:**
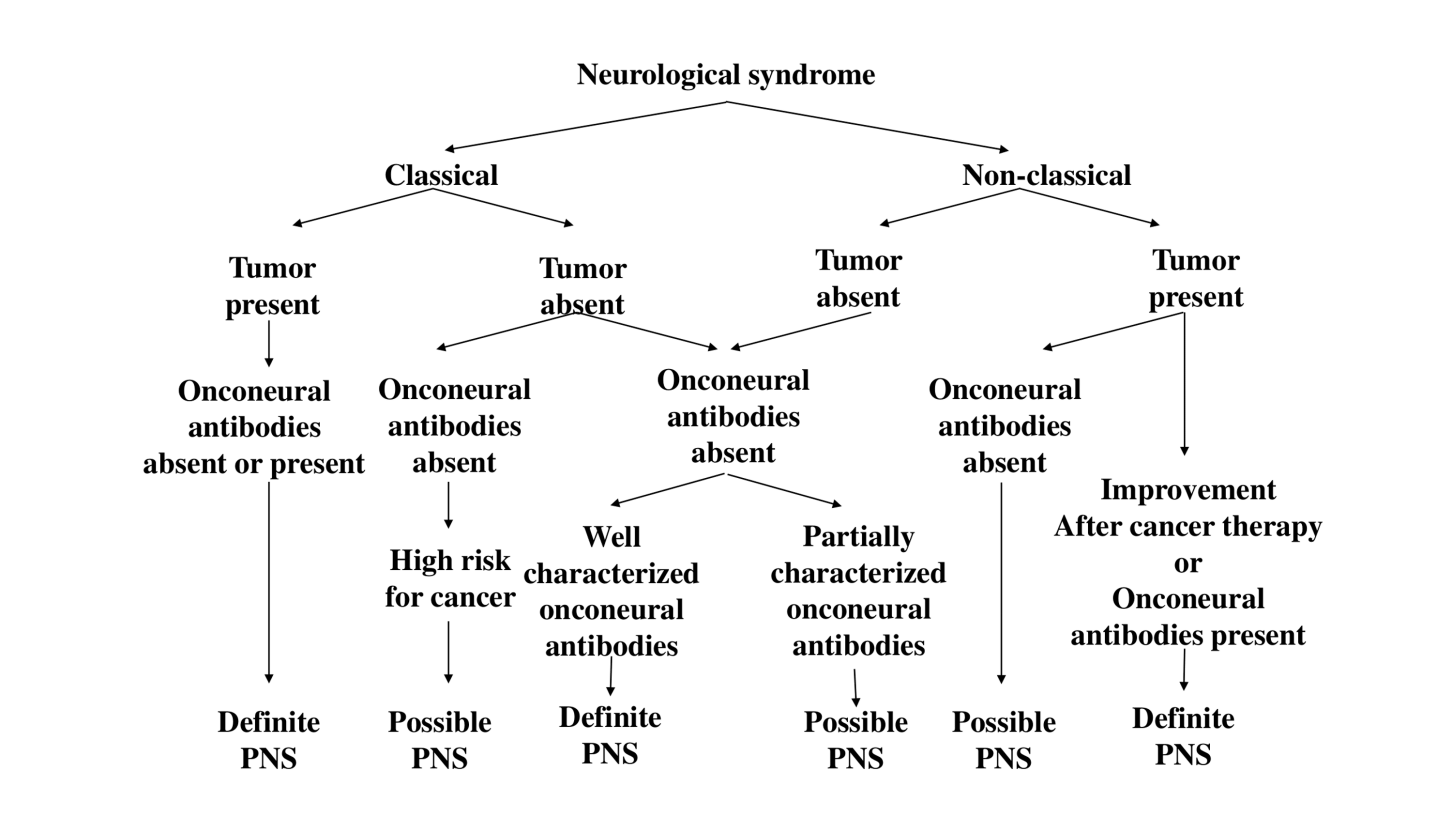
Flowchart illustrating the diagnoses of paraneoplastic neurological syndromes (PNS) in the patient. Reproduced with permission from reference [7].

In 2004, a set of recommended diagnostic criteria for PNS was established. These criteria diagnose PNS based on the presence of neurological symptoms, associated tumors, and antineuronal antibodies. Neurological symptoms are classified as either classical or nonclassical, and antineuronal antibodies are categorized into well-characterized and partially characterized onconeural antibodies. Based on these criteria, PNS is categorized as either “definite” or “possible” (Figure 3) [7]. In this case, the patient presented with subacute cerebellar symptoms, including dizziness and gait disturbance, for which MRI imaging revealed no identifiable cause. Consequently, the symptom of subacute cerebellar degeneration was classified as a hallmark of the classical syndrome. Additionally, the presence of both a parotid tumor and the Zic4 antibody supported the classification of this case as definite PNS.

The primary treatment for PNS typically involves addressing the underlying tumor through surgery, chemotherapy, and radiation with the aim of improving both oncological and neurological long-term outcomes. Additionally, the initial management of neurological symptoms often includes administering methylprednisolone, plasma exchange, or intravenous immunoglobulins to alleviate symptoms and prevent further neurological damage. In cases where these treatments are ineffective, immunosuppressants such as rituximab or cyclophosphamide can be considered [8]. Previous reports have revealed that PNS symptoms improve after the tumor is eliminated through surgery, chemotherapy, or radiation therapy [9,10]. Similarly, in our case, cerebellar symptoms resolved after radical tumor resection, supporting the findings of previous reports.

A PubMed search using the terms “paraneoplastic neurologic syndromes head and neck” identified 21 cases of PNS associated with head and neck cancers in literature published since 1969 (Table 1). Among these cases, nine involved laryngeal carcinoma, five nasopharyngeal carcinoma, three oropharyngeal carcinoma, and one each with hypopharyngeal carcinoma, nasal cavity and paranasal sinus carcinoma, tongue carcinoma, and submandibular gland carcinoma. Squamous cell carcinoma was the most common pathological type, occurring in nine of the 21 cases. Notably, no previous cases of parotid cancer have been reported, although one case of a salivary gland tumor with the same pathological type as in our case, SDC, was reported [11]. Seven cases were reported to be positive for antibodies associated with PNS, but none were positive for the Zic4 antibody.

**Table 1:** Summary of previous 21 reports of paraneoplastic neurological syndromes (PNS) with head and neck cancers and our case. NR, not reported; ND, not detected; NCPS, nasal cavity and paranasal sinus; SMG, submandibular gland; SCC, squamous cell carcinoma; NEC, neuroendocrine carcinoma; NPC, nasopharyngeal carcinoma; PGL, paraganglioma; LEC, lymphoepithelial carcinoma; SpCC, spindle cell carcinoma; SDC, salivary duct carcinoma; LEMS, Lambert-Eaton myasthenic syndrome; RT, radiotherapy; CRT, chemoradiotherapy; IVIG, intravenous immunoglobulin therapy

Age	Sex	Primary site	Histologic type	Type of PNS	Antibody	Tumour treatment	Neurological treatment	Authors	Year
NR	NR	larynx	SCC	cerebellar degeneration	NR	NR	NR	Muller E [13]	1969
58	M	larynx	SCC	LEMS	NR	surgery	NR	Fontanel J [14]	1973
64	F	larynx	NEC	LEMS	NR	CRT	NR	Medina J [15]	1984
58	M	larynx	SCC	LEMS	NR	CRT	NR	Ferroir J [16]	1989
66	M	larynx	SCC	ataxia	NR	surgery	NR	Garcia F [17]	1998
74	M	larynx	SCC	encephalomyelitis	anti-Hu	RT	none	Baijens L [18]	2006
39	M	nasopharynx	NPC	inflammatory myelopathy	NR	RT, chemotherapy	IVIG, steroids	Chan KH [19]	2007
64	M	larynx	SCC	LEMS	NR	surgery	IVIG	Shipley E [20]	2008
60	F	tongue	SCC	cerebellar degeneration	anti-CV2	CRT	none	Emmanouel S [21]	2010
49	F	hypopharynx	PGL	encephalomyelitis	NR	surgery	steroids	Huiqin T [22]	2013
38	M	oropharynx	LEC	cerebellar degeneration	ND	surgery	steroids	Henke C [23]	2013
50	F	nasopharynx	NPC	opsoclonus-myoclonus	NR	CRT	steroids, IVIG	Bilal G [24]	2015
60	F	NCPS	SpCC	cerebellar degeneration	anti-Hu	RT, surgery, CRT	none	Florian H [25]	2015
53	M	larynx	SCC	encephalomyelitis	NR	NR	NR	Erro A [26]	2016
68	M	oropharynx	SCC	cerebellar degeneration	ND	CRT	none	Yong L [27]	2017
76	M	nasopharynx	NPC	sensory and motor neuropathy	NR	RT	none	Ng SY [28]	2017
78	M	oropharynx	NEC	LEMS	VGCC	CRT	none	Takasugi J [29]	2018
46	M	nasopharynx	NPC	cerebellar degeneration	anti-Yo	CRT	IVIG, steroids	Bhardwaj S [30]	2019
68	M	larynx	NEC	LEMS	NR	surgery, CRT	NR	Mesolella M [31]	2021
50s	M	nasopharynx	LEC	cerebellar degeneration	anti-RI	CRT	IVIG, steroids	Kristen E [5]	2022
60	F	SMG	SDC	cerebellar degeneration	anti-Yo	surgery	IVIG, steroids	Takeshi I [11]	2022
66	F	parotid gland	SDC	cerebellar degeneration	anti-Zic4	surgery, RT	steroids	our case	

Bataller et al. reported 61 cases of adult patients with the Zic4 antibody, of which 49 were diagnosed with PNS. Among these 49 patients, nine were positive only for the Zic4 antibody, without the concurrent presence of other onconeural antibodies. These patients showed a higher tendency to develop cerebellar dysfunction. In our case, the patient tested positive only for the Zic4 antibody and presented with cerebellar symptoms, which is consistent with previous reports. Of the 61 patients with Zic4 antibody, 44 were associated with small cell lung cancer, the highest number observed, while no cases with head and neck cancer were reported [12]. Our case represents the first reported instance of Zic4 antibody-positive PNS associated with head and neck cancer.

## Conclusions

We presented the first reported case of PNS attributed to parotid cancer expressing the Zic4 antibody alongside a review of 21 previous cases of PNS associated with head and neck cancer. Despite their rarity, understanding the relationship between PNS and head and neck cancers is crucial for timely and accurate diagnosis. Early identification of PNS can provide valuable clues about the presence of underlying tumors, leading to an improved prognosis.
